# Genomic characteristics and preliminary study on *in vitro* infection properties of canine parvovirus type 2c derived from a canine anal swab sample

**DOI:** 10.3389/fvets.2026.1938079

**Published:** 2026-10-30

**Authors:** Dachao Mou, Yanqiong Chen, Shasha Wu, Jian Huang, Yin Guo, Xiufeng Bai

**Affiliations:** 1 Laboratory of Human Diseases and Immunotherapy, West China Hospital, Sichuan University, Chengdu, China; 2 Laboratory of Clinical Veterinary Medicine, College of Animal Science and Veterinary Medicine, Southwest Minzu University, Chengdu, China; 3 Chengdu Zhonghuan Animal Hospital Co., Ltd, Chengdu, China

**Keywords:** canine parvovirus, complete genomenear-complete genome, CPV-2c, *in vitro* infection, MDC-CPV, phylogeny, VP2

## Abstract

**Objective:**

This study aimed to determine the genomic characteristics and genotype of a CPV isolate (MDC-CPV) recovered from a vaccinated dog, and to investigate its *in vitro* infectivity and replication features in A72 and CRFK cell lines.

**Methods:**

Clinical samples were subjected to nucleic acid detection for common canine pathogens and CPV antigen testing. Sequencing was performed using the Illumina NovaSeq PE150 platform. Following quality control, assembly, and annotation, the near-complete genome sequence of MDC-CPV was obtained. Homology searches were conducted using BLASTn. Genotyping was determined based on key amino acid residues in VP2, and phylogenetic analyses were performed using both the full-length VP2 coding sequence (CDS) and the whole-genome reference sequences. *In vitro* infection was subsequently validated in A72 and CRFK cells. Viral infection-associated characteristics were evaluated by observing cytopathic effects (CPE), and through Western blot, immunofluorescence, qPCR, TCID50 assay, and transmission electron microscopy.

**Results:**

The genome length of MDC-CPV was 5,358 nucleotides, with no ambiguous bases identified. Genome annotation revealed that MDC-CPV contains typical CPV coding regions, including NS1, NS2, VP1, and VP2. The VP2 CDS was 1755 nucleotides in length, encoding 584 amino acids. Key VP2 residues for MDC-CPV were 297Ala/426Glu, along with a combination of other residues (87Leu, 101Thr, 300Gly, 305Tyr, 324Ile, 375Asp, 555Val, 564Ser, and 568Gly), supporting its classification as CPV-2c. Phylogenetic analysis placed MDC-CPV within the CPV-2c clade, showing close genetic relatedness to several CPV-2c reference strains from China and other regions in East Asia. In *in vitro* infection assays, CPE was observed in both A72 and CRFK cells inoculated with MDC-CPV. Furthermore, CPV-related protein expression and virus-like particles were detected. Quantitative PCR and TCID50 assays in CRFK cells demonstrated productive replication, with viral DNA levels and infectious virus titers increasing substantially from 48 h post-infection, reaching peak titers of approximately 5.8 log10 TCID50/1 mL at 96 h post-infection.

**Conclusion:**

MDC-CPV is a CPV-2c strain isolated from a clinical sample of a vaccinated dog. Its genomic and VP2 genotypic characteristics are consistent with recently circulating CPV-2c strains. These findings provide a reference for molecular epidemiological surveillance of CPV-2c in the Sichuan region and for research on CPV infection in the context of vaccination.

## 1 Introduction

Canine parvovirus (CPV) is one of the important pathogens causing acute hemorrhagic enteritis in dogs and severe infections in puppies (1). CPV belongs to the genus Protoparvovirus within the family Parvoviridae, and the related parvoviruses infecting dogs, cats, and mustelids are currently classified as Carnivore protoparvovirus 1. The virus is a non-enveloped, single-stranded DNA virus with a genome length of approximately 5 kb, primarily encoding the non-structural proteins NS1/NS2 and the structural proteins VP1/VP2. Since the emergence and rapid spread of CPV-2 in the late 1970s, the virus has undergone continuous evolution within the canine population, resulting in antigenic variants such as CPV-2a, CPV-2b, and CPV-2c (2, 3). VP2 is the major capsid protein of CPV and is involved in receptor binding, host range determination, antigenic variation, and viral evolution. Amino acid residue 426 of VP2 is commonly used as a key site to distinguish CPV-2a, CPV-2b, and CPV-2c, corresponding to Asn/N, Asp/D, and Glu/E, respectively (3). In addition to residue 426, other sites such as VP2 residues 297, 300, 305, 324, 375, 555, 564, and 568 are also frequently used to characterize the genetic background and evolutionary features of viral strains. In recent years, although multiple CPV-2c strains have been reported domestically and internationally, full-genome reports of CPV-2c originating from dogs with a complete history of vaccination remain limited. Furthermore, comprehensive analyses integrating cellular infection characteristics are lacking (4, 5). This viral type can be detected in dogs with a history of vaccination; however, the detection of CPV-2c in a single case cannot be directly equated to vaccine failure. Its occurrence may be associated with multiple factors, including the vaccination regimen, interference from maternal antibodies, the individual immune status, the viral exposure dose, and antigenic differences among circulating strains (6). Therefore, conducting whole-genome sequencing, VP2 genotyping, phylogenetic analysis, and *in vitro* infectivity validation of CPV isolates from clinical samples is conducive to understanding the molecular characteristics and potential biological differences of regional epidemic strains. In this study, CPV was detected in a clinical sample obtained from a dog with a history of CPV vaccination in Chengdu, Sichuan Province, and its genome sequence was obtained and designated as MDC-CPV. This study systematically analyzed its genome structure, key VP2 residues, and phylogenetic relationships, and preliminarily evaluated its infection characteristics in A72 and CRFK cells using *in vitro* infection experiments, we aim to comprehensively characterize the genomic features and *in vitro* infectivity of a CPV-2c strain isolated from a vaccinated dog in the Sichuan region. These findings provide fundamental data for the continuous monitoring of CPV-2c and subsequent biological studies.

## 2 Materials and methods

### 2.1 Sample source and clinical information

The MDC-CPV sample was collected from a 5-month-old male Toy/Miniature Poodle in Chengdu, Sichuan Province, China. The dog had been vaccinated with a canine tetravalent vaccine containing canine distemper virus, canine parainfluenza virus, canine adenovirus, and canine parvovirus in December 2025, January 2026, and February 2026, with all antibody tests yielding positive results. Subsequently, the dog developed clinical signs including vomiting, depression, and weight loss, but no overt diarrhea was observed. The sample was collected on March 28, 2026, using an anal swab. All sample collection and testing procedures were performed with the informed consent of the animal owner and in compliance with the ethical and biosafety requirements of the host institution. Sample collection was performed with the owner’s written informed consent. No experimental animal procedures were conducted; the sample was obtained as part of routine clinical diagnostic testing (Table 1).

**Table 1:** Sequencing information of the MDC-CPV sample.

Item	Content
Sample/strain ID	MDC-CPV
Host	Canine (*Canis lupus familiaris*), 5-month-old male Toy/Miniature Poodle
Sampling location/date	Chengdu, Sichuan, China; March 28, 2026
Sample type	Anal swab
Clinical manifestations	Vomiting, depression, weight loss, leukopenia; no apparent diarrhea
Vaccination history	Vaccinated with a canine tetravalent vaccine (KeWangFu) on December 20, 2025, January 10, 2026, and February 2, 2026
Sequencing platform	Illumina NovaSeq PE150
Genome length	5,358 nt
GenBank accession number	PZ493559
Preliminary genotype	CPV-2c(VP2 297A/426E)

nt, nucleotides; CPV, canine parvovirus; VP2, viral protein 2.

### 2.2 Detection of pathogen nucleic acids and antigens

Total nucleic acids were extracted from clinical specimens in accordance with the manufacturer’s instructions provided in the respective kit. Polymerase chain reaction (PCR) or reverse transcription PCR (RT-PCR) assays were performed for the detection of canine parvovirus type 2 (CPV-2), canine coronavirus (CCoV), canine distemper virus (CDV), canine adenovirus types 1 and 2 (CAdV-1/2), canine parainfluenza virus (CPIV), canine herpesvirus type 1 (CaHV-1/CHV-1), *Bordetella bronchiseptica*, and pathogenic Leptospira spp. Nucleic acids were extracted using FastPure DNA Extraction Kit (Vazyme DC510). PCR reactions were performed in a final volume of 25 μL containing 2 x PCR mix (Vazyme). Each run included an appropriate positive control, and a no-template control. The reverse transcription kit was purchased from Chengdu Jinsha Biotechnology, and the 2 × PCR master mix was obtained from Chengdu Rongwei Gene Technology. For CPV, a commercial colloidal gold rapid immunochromatographic test strip was additionally employed for preliminary antigen screening (Table 2).

**Table 2:** PCR primers used in this study.

Pathogen	Method	Target name	Product length	Forward primer 5′ → 3′	Reverse primer 5′ → 3′	Reference
CPV-2	PCR	*NS1* gene	125 bp	CTCCTCTGACTCCGGACGTA	CGCTTGCACGTCTTTGTGAG	(7)
CCoV	RT-PCR	*M* gene	409 bp	TCCAGATATGTAATGTTCGG	TCTGTTGAGTAATCACCAGCT	(8)
CDV	RT-PCR	*NP*/*N* gene	290 bp	AACAGRRATTGCTGAGGACYTAT	TCCARRRATAACCATGTAYGGTGC	(9)
CAdV-1/CAdV-2	PCR	*Hexon* gene	CAdV-1: 508 bp; CAdV-2: 1030 bp	CGCGCTGAACATTACTACCTTGTC	CCTAGAGCACTTCGTGTCCGCTT	(10)
CPIV	RT-PCR	*NP* gene	187 bp	GCCGTGGAGAGATCAATGCCTAT	GCGCAGTCATGCACTTGCAAGT	(11)
CaHV-1/CHV-1	PCR	*Glycoprotein B, gB*/*GB* gene	136 bp	ACAGAGTTGATTGATAGAAGAGGTATG	CTGGTGTATTAAACTTTGAAGGCTTTA	(11)
*Bordetella bronchiseptica*	PCR	*fla*/*flagellin* gene	237 bp	TGGCGCCTGCCCTATC	AGGCTCCCAAGAGAGAAA	(12)
*Pathogenic Leptospira* spp.	PCR	*lipL32* gene	423 bp	CGCTGAAATGGGAGTTCGTATGATT	CCAACAGATGCAACGAAAGATCCTTT	(13)

CPV-2, canine parvovirus type 2; CCoV, canine coronavirus; CDV, canine distemper virus; CAdV-1/2, canine adenovirus type 1/2; CPIV, canine parainfluenza virus; CaHV-1/CHV-1, canine herpesvirus 1. NS1, non-structural protein 1; M, membrane protein; NP, nucleoprotein; Hexon, hexon protein; gB, glycoprotein B; fla, flagellin gene; lipL32, outer membrane lipoprotein L32. R = A/G; Y = C/T, degenerate bases in primers. bp, base pair.

### 2.3 Whole-genome sequencing, assembly, and annotation

Following viral nucleic acid extraction from CPV-positive samples, Paired-end sequencing was performed on the Illumina NovaSeq platform using a PE150 strategy. A total of 12,718,320 raw reads (6,359,160 read pairs), corresponding to 1,907,748,000 bases, were generated. The raw dataset showed Q20 and Q30 rates of 96.22 and 92.92%, respectively. Raw reads were quality filtered using fastp v0.23.4. Adapter/low-quality trimming and read correction were performed using the parameters -5 -3 -q 20 -c -l 50. After quality filtering, 12,404,882 reads (6,202,441 read pairs; 1,748,135,708 bases) remained, with Q20 and Q30 rates increasing to 97.39 and 94.36%, respectively. The mean lengths of both R1 and R2 reads after filtering were approximately 140 bp. rRNA, host-derived, and bacterial contaminating sequences were subsequently removed. Following rRNA filtering, 6,172,876 read pairs remained; 140,436 read pairs remained following host-sequence filtering, and 50,375 read pairs remained following further removal of bacterial sequences. *De novo* assembly was performed using SPAdes v3.14.1 and SOAPdenovo v2.04, followed by identification of virus-associated contigs using BLAST+ v2.10.0 against viral/nucleotide databases. The principal CPV-associated contig was 5,358 nt in length. Its closest BLAST match was canine parvovirus strain CPV-SH1516 (GenBank accession MG013488.1), with 99.812% nucleotide identity over a 4,795-bp alignment. Sequencing reads were mapped back to the assembled viral contig to assess assembly support. The 5,358-nt MDC-CPV contig showed an average depth of 52.94×, a median depth of 53×, and 100% nucleotide coverage. A total of 98.99% of positions were covered at ≥10×, whereas 5.41% were covered at ≥100×. Genome annotation was performed using Prokka v1.14.5, followed by manual/reference-based verification of the major CPV coding regions. Genome annotation was performed using SnapGene and ORF Finder, in conjunction with closely related CPV reference sequences obtained from GenBank. Particular attention was devoted to verifying the coding regions of NS1, NS2, VP1, and VP2.

### 2.4 Homology search and VP2 typing

The near-complete genome sequence of MDC-CPV was submitted to the NCBI BLASTn database for homology search. The top 30 closely related sequences were recorded, including their accession numbers, sequence names, countries/regions, isolation years, query cover, and percent identity. Furthermore, the VP2 coding sequence (CDS) of MDC-CPV was extracted and translated into an amino acid sequence. Key amino acid residues at positions 297, 300, 305, 324, 375, 426, 555, 564, and 568 of the VP2 protein were analyzed. The identification of CPV-2c was primarily based on the presence of glutamic acid (Glu/E) at position 426 of VP2, in combination with other relevant residues such as alanine (Ala/A) at position 297, allowing for a comprehensive determination.

### 2.5 Phylogenetic and sequence similarity analysis

Representative sequences of CPV-2, CPV-2a, CPV-2b, CPV-2c, and FPV were downloaded from GenBank, and a reference dataset was constructed by incorporating the MDC-CPV strain. Multiple sequence alignment of the full-length VP2 coding sequences (CDS) was performed using MAFFT. After manual inspection and necessary trimming, maximum likelihood phylogenetic analysis was conducted using IQ-TREE. The best-fit nucleotide substitution model was selected by ModelFinder based on the Bayesian Information Criterion (BIC). Node support was assessed using 1,000 SH-aLRT replicates and 1,000 ultrafast bootstrap replicates. Sequence similarity matrices were calculated and visualized using SDT or an equivalent tool. For whole-genome phylogenetic analysis, only complete or nearly complete genome sequences with comparable length and annotation quality were included (Table 3).

**Table 3:** Genomic information of different CPV subtypes used in this study.

Accession number	Subtype	Country/region	Genome length (nt)	Isolation date
OR230516.1	CPV-2a	Brazil	5,376	2022
OR230515.1	CPV-2a	Brazil	5,367	2022
OR230514.1	CPV-2a	Brazil	5,376	2022
OR230513.1	CPV-2a	Brazil	5,370	2022
OR230512.1	CPV-2a	Brazil	5,369	2022
OR230511.1	CPV-2a	Brazil	5,376	2022
OR230510.1	CPV-2a	Brazil	5,351	2022
MT629886.1	CPV-2a	—	4,840	—
OP972596.1	CPV-2a	—	5,001	—
MW539053.1	CPV-2b	—	4,873	—
ON733252.1	CPV-2b	—	5,016	2021
MW653252.1	CPV-2b	Iran	4,810	—
MW653251.1	CPV-2b	Iran	5,020	—
JQ268284.1	CPV-2b	—	5,053	—
KY073269.1	CPV-2c	—	5,318	—
MN832850.1	CPV-2c	Taiwan, China	4,960	2018
MH660909.1	CPV-2c	—	5,075	—
PP973493.1	FPV	—	5,123	—
OR198066.1	FPV	—	4,903	1980
MT614366.1	FPV	—	5,125	—
M38245.1	CPV-2	—	5,124	—
PZ493559	CPV-2c	Sichuan, China	5,358	2026

FPV, feline panleukopenia virus; “—” indicates that the relevant information was not available in the GenBank record.

### 2.6 Cell culture and virus isolation/infection

Virus culture was performed in the Animal Medicine Laboratory (BSL-2) at Southwest Minzu University. Anal swab samples were diluted in phosphate-buffered saline (PBS); a portion was used for pathogen detection by polymerase chain reaction (PCR) and colloidal gold immunochromatography, while the remaining sample was centrifuged at 12,000 × g and 4 °C for 5 min to remove debris. The supernatant was filtered through a 0.22 μm filter and temporarily stored at −20 °C. A72 and CRFK cells were revived and cultured in Dulbecco’s Modified Eagle Medium (DMEM) supplemented with 10% fetal bovine serum (FBS) and 1% penicillin–streptomycin, and maintained at 37 °C in a 5% CO₂ atmosphere. When the cells reached approximately 80% confluence, they were inoculated with processed MDC-CPV-positive samples or virus passage fluids. This passaged virus stock was used for the subsequent *in vitro* infection assays described in this study. After viral adsorption, the inoculum was replaced with maintenance medium, and the cells were monitored for cytopathic effects (CPE) at various time points. Cells not inoculated with virus served as negative controls.

### 2.7 Western blot, immunofluorescence, and transmission electron microscopy

Cells were collected 24 h post-infection with canine parvovirus (CPV) for the detection of VP2 protein expression via Western blot analysis. Protein samples were separated by SDS-PAGE and transferred onto membranes, followed by incubation with an anti-VP2 (CPV) antibody (MCE-HY-P86755) and the corresponding secondary antibody (HUABIO-HA1006). GAPDH served as the loading control. For immunofluorescence assays, CPV-infected cells were fixed, permeabilized, and blocked, then incubated with the anti-VP2 (CPV) antibody, followed by incubation with a fluorescence-conjugated secondary antibody. Nuclei were counterstained with DAPI, and the cells were visualized under a fluorescence microscope. For transmission electron microscopy, supernatants from CRFK cells infected with CPV for 24 h were collected, negatively stained, and examined for the morphology of virus-like.

### 2.8 Quantification of MDC-CPV replication kinetics by quantitative PCR

To evaluate the replication kinetics of MDC-CPV *in vitro*, CRFK cells were inoculated with MDC-CPV as described above, while mock-infected CRFK cells were processed in parallel as negative controls. Samples were collected at 0, 24, 48, 72, 96, and 120 h post-infection (hpi). CPV DNA was quantified using a SYBR Green-based quantitative PCR assay targeting a conserved region of the viral NS gene. The primer sequences were NS-CPV-DEG-F (5′-CGCTTGCACGTCTTTGTGAG-3′) and NS-CPV-DEG-R (5′-CTCCTCTGACTCCGGACGTA-3′), generating a 125-bp amplicon.

### 2.9 Determination of viral titers and growth kinetics by TCID50 assay

To determine the production of infectious progeny virus, culture supernatants from the third passage of MDC-CPV were collected for TCID50 determination. Virus-containing samples were collected from MDC-CPV-infected CRFK cell cultures at 0, 24, 48, 72, 96, and 120 h post-infection (hpi). Samples were serially diluted tenfold in maintenance medium and inoculated onto CRFK cell monolayers cultured in 96-well plates. CRFK cells were seeded at 2 × 10^4 cells per well in a volume of 100 μL, and 100 μL of each tenfold virus dilution was added to the corresponding wells. Replicate wells were used for each dilution. Following viral adsorption for 2 h, the inoculum was removed and replaced with maintenance medium, or handled according to the specific experimental protocol. The cells were subsequently incubated at 37 °C in a humidified atmosphere containing 5% CO₂ and monitored for CPV-associated cytopathic effects until the assay endpoint was reached. The 50% tissue culture infectious dose (TCID50) was calculated using the Reed–Muench method and expressed as log10 TCID50/mL. Viral titers determined at the indicated time points were used to construct the *in vitro* growth curve of MDC-CPV in CRFK cells. All experiments were performed independently three times.

### 2.10 Recombination and phylogenetic analyses

Near-complete CPV genome sequences were aligned using MAFFT version 7.525 with the --auto option. Alignment columns represented in fewer than 70% of the sequences were excluded to minimize the influence of incomplete genome termini, resulting in a 4,680-nt near-complete whole-genome alignment. The NS1 and VP2 coding-region alignments comprised 2,007 and 1,755 nucleotides, respectively. Maximum-likelihood phylogenies were reconstructed using IQ-TREE version 2.0.7. The best-fitting nucleotide substitution model was selected using ModelFinder, and branch support was assessed using 1,000 SH-aLRT replicates and 1,000 ultrafast-bootstrap replicates. The selected models were TN + F + R2 for the whole-genome and NS1 datasets and HKY + F + R2 for the VP2 dataset. Potential recombination involving MDC-CPV was evaluated using OpenRDP version RDP5 5.93. The RDP, GENECONV, BootScan, MaxChi, Chimaera, SiScan, and 3Seq methods were applied to an MDC-CPV-focused panel consisting of MDC-CPV and its 40 closest non-identical reference sequences. Because of its substantially greater computational demand, the completed SiScan sensitivity analysis used MDC-CPV and 14 representative candidate parental sequences, with 100 *p*-value permutations and 20 scan permutations. Statistical significance was defined as a Bonferroni-corrected *p* value < 0.05. A recombination event was regarded as well supported only when a concordant breakpoint region was detected by at least three independent methods.

## 3 Results

### 3.1 Detection of CPV positivity in clinical samples

Common canine pathogen nucleic acid screening was performed on the MDC-CPV clinical sample. PCR results revealed an amplification band at the CPV-2 target, consistent with the expected size, whereas no definitive positive results were obtained for CCoV, CDV, CAdV-1/2, CPIV, CaHV-1/CHV-1, *B. bronchiseptica*, and pathogenic Leptospira spp. A colloidal gold rapid test also indicated CPV antigen positivity. These findings suggested the presence of CPV infection signals in the case sample, thereby providing a basis for subsequent whole-genome sequencing and *in vitro* infection (Figure 1).

**Figure 1:**
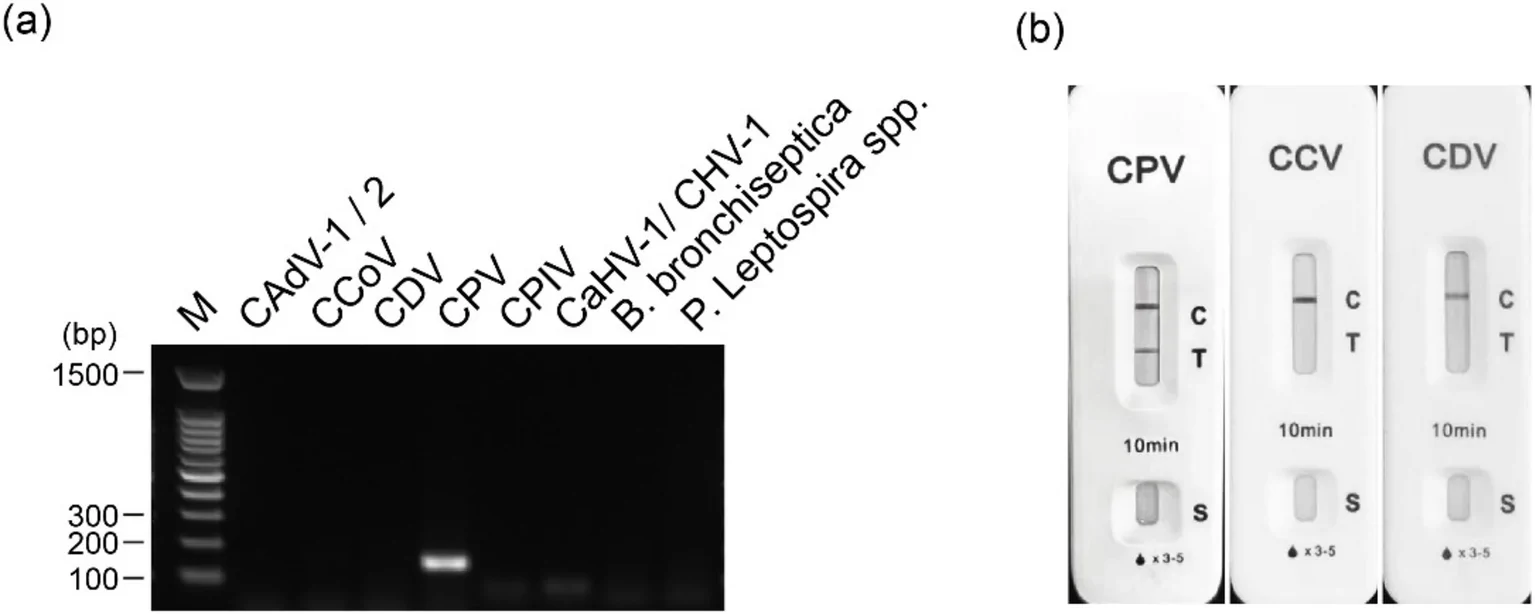
Preliminary etiological screening of MDC-CPV clinical samples. **(a)** PCR/RT-PCR detection results for different canine pathogens; **(b)** colloidal gold rapid test results for common canine pathogens.

### 3.2 MDC-CPV genome structure

Following sequencing, assembly, and annotation, the complete genome sequence of MDC-CPV was obtained, with a total length of 5,358 nucleotides (nt) and no ambiguous bases. The nucleotide composition was determined to be A (1,912), C (842), G (1,078), and T (1,526), with a high A + T content, consistent with the genomic characteristics of single-stranded DNA viruses within the Parvoviridae family. Genome annotation revealed that MDC-CPV contains the typical coding regions of canine parvovirus (CPV), including NS1, NS2, VP1, and VP2. The VP2 coding sequence (CDS) is located at nucleotide positions 2,621–4,375, comprising 1,755 nucleotides, which encode a protein of 584 amino acids (Table 4).

**Table 4:** Genomic annotation information of MDC-CPV.

Gene/Feature	Type	Coordinates	Nucleic acid/Amino acid length
NS1	CDS	107–2113	2004 nt; 668 aa
NS2	CDS	107–367; 1840–2076	—
VP1	CDS	2120–2151; 2224–4,375	—
VP2	CDS	2621–4375	1752 nt; 584 aa

CDS, coding sequence; aa, amino acids. NS, non-structural protein; VP, viral capsid protein.

### 3.3 Homology search and whole-genome sequence relationship

BLASTn homology analysis revealed that MDC-CPV shares a high degree of similarity with multiple CPV-2c sequences deposited in GenBank, with the highest nucleotide identity reaching approximately 99.81%. The top 30 related sequences were primarily derived from CPV-2c or related canine parvovirus strains reported in China and other East Asian regions. Based on query coverage and percent identity, MDC-CPV is closely related at the whole-genome level to recently circulating CPV-2c strains (Figure 2; Table 5).

**Figure 2:**
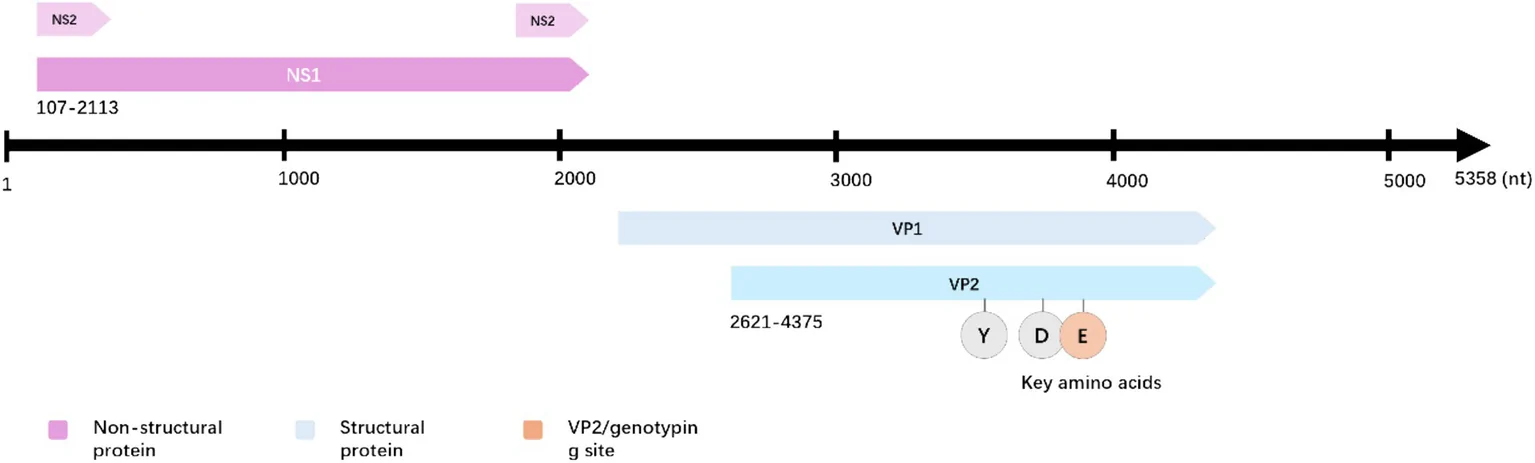
Schematic diagram of the MDC-CPV genomic structure.

**Table 5:** Top 30 BLASTn matches for the complete genomic sequence of MDC-CPV.

No.	Accession number	Strain	Country/Region	Isolation year	Query coverage (%)	Homology (%)	Sequence length (nt)
1	MG013488	CPV-SH1516	Shanghai, China	2017	89.44	99.81	5,059
2	MH476587	Canine/China/18/2017	China	2017	89.44	99.79	5,051
3	MH476583	Canine/China/14/2017	China	2017	89.44	99.79	5,053
4	MT648203	CPV-AHhf27	Anhui, China	2019	89.44	99.79	5,058
5	MN832850	Taiwan/2018	Taiwan, China	2018	89.44	99.79	4,960
6	MT648207	CPV-AHmas9	Anhui, China	2018	89.44	99.77	5,061
7	MT010564	CPV-AHhf1	Anhui, China	2018	89.44	99.77	5,061
8	MT648205	CPV-mas2	—	2018	89.44	99.77	5,055
9	MW650830	CPV-SH2001	Shanghai, China	2020	89.44	99.75	5,005
10	PV784947	CPV-KZ-2024	Kazakhstan	2024	89.44	99.75	5,047
11	MH476584	Canine/China/15/2017	China	2017	89.44	99.73	5,053
12	MH476581	Canine/China/12/2017	China	2017	89.44	99.73	5,054
13	MT648204	CPV-AHhf28	Anhui, China	2019	89.44	99.73	5,058
14	MH660909	5 MGL	-	2017	89.44	99.73	5,075
15	MH476592	Canine/China/23/2017	China	2017	89.44	99.71	5,052
16	MF805796	Canine/China/08/2016	China	2016	89.44	99.71	5,051
17	MH476585	Canine/China/16/2017	China	2017	89.44	99.71	5,051
18	MT648210	CPV-AHcf4	Anhui, China	2019	89.44	99.71	5,061
19	MT394167	QD-20	Shandong, China	—	89.44	99.67	5,058
20	MF805789	Canine/China/01/2016	China	2016	89.44	99.65	5,051
21	MF805792	Canine/China/04/2016	China	2016	89.44	99.65	5,051
22	MF805797	Canine/China/09/2016	China	2016	89.44	99.65	5,051
23	MF805795	Canine/China/07/2016	China	2016	89.44	99.65	5,051
24	MT394165	QD-202	Shandong, China	—	89.44	99.65	5,055
25	MT648202	CPV-AHhf7	Anhui, China	2019	89.44	99.65	5,058
26	MT892649	China-XY	China	2019	89.44	99.58	5,059
27	MT394166	QD-201	Shandong, China	—	89.44	99.31	5,058
28	OQ198119	ParvoviridaeDogfe435C1	—	2020	88.24	99.64	4,795
29	PQ180794	Hebei_129	Hebei, China	2023	87.91	99.75	4,749
30	OR528752	CPV_PE1_2c	—	2017	89.44	99.10	5,061

Query coverage, the percentage of the query sequence aligned to the subject sequence in BLASTn; Homology, nucleotide identity between MDC-CPV and the reference sequence.

### 3.4 Key amino acid residues in VP2 support the classification of MDC-CPV as CPV-2c

The VP2 coding sequence (CDS) of MDC-CPV is 1,755 nucleotides (nt) in length, encoding 584 amino acids (aa). Analysis of key residues revealed that the amino acid at position 426 of the MDC-CPV VP2 protein is glutamic acid (Glu/E), which is a critical marker for the classification of CPV-2c. In addition to Glu at position 426, MDC-CPV harbored a combination of other residues, including Leu (L) at position 87, Thr (T) at 101, Ala (A) at 297, Gly (G) at 300, Tyr (Y) at 305, Ile (I) at 324, Asp (D) at 375. Comparison with the representative sequences of CPV-2, CPV-2a, and CPV-2b demonstrated that MDC-CPV shared identity with CPV-2c at the key typing residues (Figure 3; Table 6).

**Figure 3:**
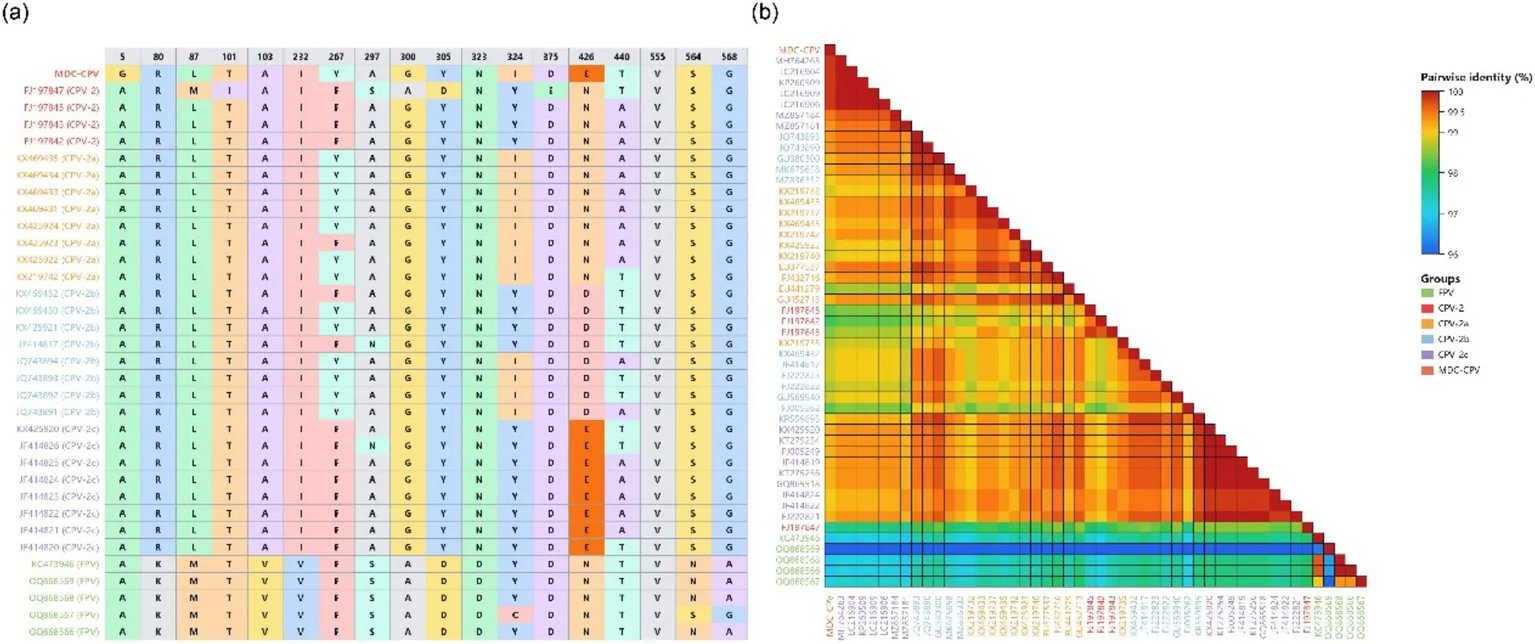
Comparison of VP2 homology and critical amino acid residues between MDC-CPV and CPV reference sequences.

**Table 6:** Comparison of key VP2 sites between MDC-CPV and different CPV subtypes.

Sequence/Subtype	VP2-297	VP2-426	Other representative sites	Subtype determination
CPV-2 Reference Strain	A/S	N	Traditional CPV-2 related site combination	CPV-2
CPV-2a Reference Strain	A/N/S	N	Novel CPV-2a-related site combination	CPV-2a
CPV-2b Reference Strain	A/S/N	D	CPV-2b-related site combination	CPV-2b
CPV-2c Reference Strain	A/N	E	CPV-2c-related site combination	CPV-2c
MDC-CPV	A	E	87L, 101T, 300G, 305Y, 324I, 375D, 555V, 564S, 568G	CPV-2c

Amino acids are indicated by single-letter abbreviations: A, alanine; N, asparagine; S, serine; D, aspartic acid; E, glutamic acid; L, leucine; T, threonine; G, glycine; Y, tyrosine; I, isoleucine; V, valine. The numbers represent amino acid positions in the VP2 protein.

### 3.5 Phylogenetic analysis of VP2

To further elucidate the evolutionary relationships between MDC-CPV and reference sequences representing different CPV genotypes, a maximum likelihood phylogenetic tree was constructed based on the full-length VP2 coding sequence (CDS). The reference sequence dataset included representative strains of CPV-2, CPV-2a, CPV-2b, and CPV-2c, with FPV-related sequences used as an outgroup. The best-fit substitution model was selected using IQ-TREE Model Finder based on the Bayesian Information Criterion (BIC). Branch support was assessed using 1,000 replicates of SH-aLRT and 1,000 replicates of ultrafast bootstrap. The results demonstrated that MDC-CPV clustered within the CPV-2c clade, grouping with multiple recent CPV-2c reference sequences, further supporting its classification as the CPV-2c genotype. No well-supported recombination event involving MDC-CPV was detected. Signals generated by individual methods were not corroborated by at least three independent algorithms and did not define a consistent overlapping breakpoint interval. GENECONV, Chimaera, SiScan, and 3Seq produced no evidence of an MDC-CPV recombination event. The isolated signals identified by RDP, BootScan, or MaxChi were method-specific or associated with alignment-terminal regions and were therefore not considered reliable evidence of recombination. The whole-genome, NS1, and VP2 maximum-likelihood trees consistently placed MDC-CPV within the CPV-2c lineage. MDC-CPV clustered most closely with PZ569219.1 in the whole-genome and VP2 trees, with SH-aLRT/ultrafast-bootstrap support values of 75.6/90 and 84/97, respectively. Its nearest sequence in the NS1 tree was PZ529563.1, but this association was unsupported (0/40). Overall, the phylogenetic placement of MDC-CPV was concordant across the three datasets (Figure 4; Table 7).

**Figure 4:**
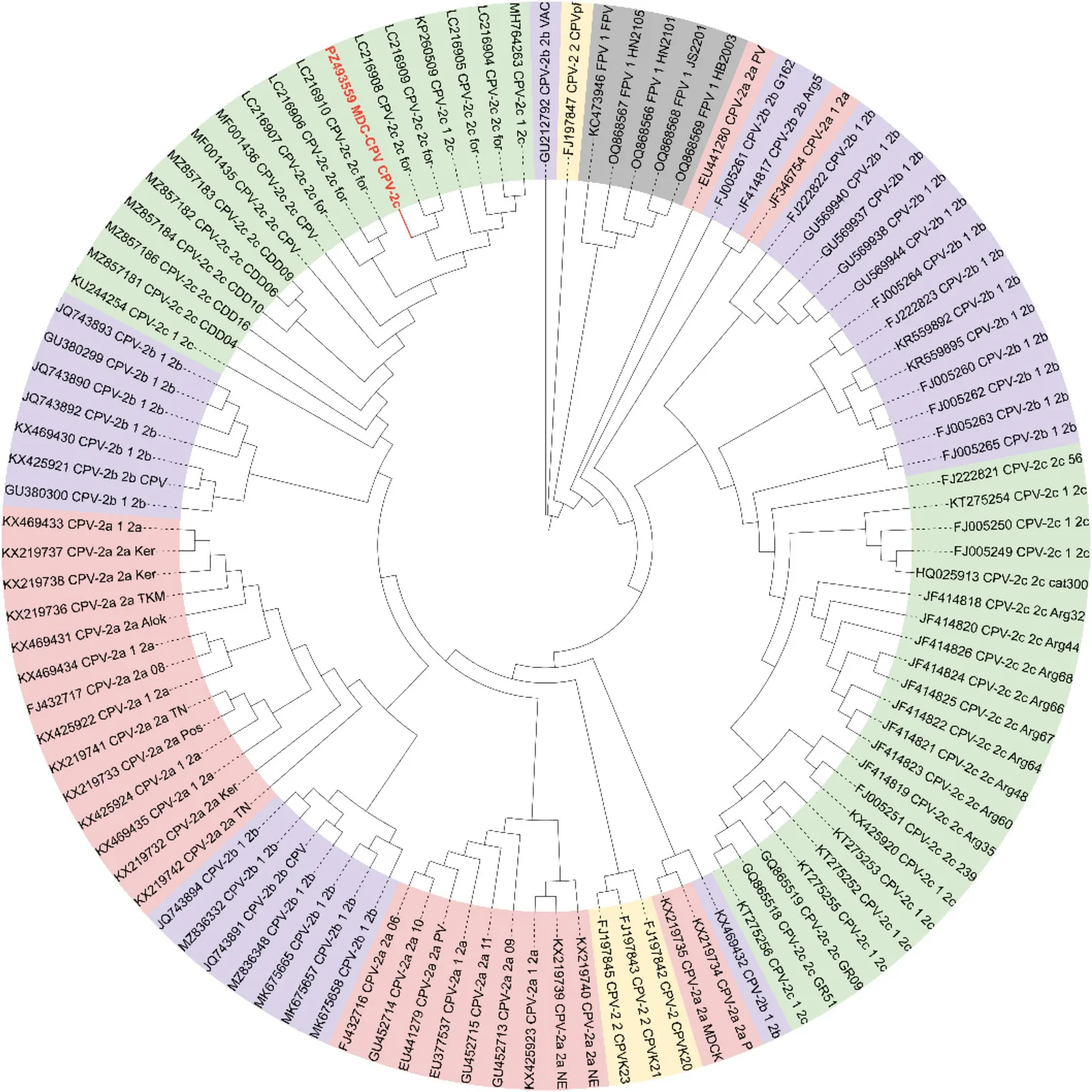
Maximum likelihood phylogenetic tree constructed from full-length VP2 CDS sequences. MDC-CPV is highlighted in red.

**Table 7:** Representative amino-acid signatures of MDC-CPV in structural and non-structural proteins.

Protein	Residue	MDC-CPV	Interpretation
NS1	60	V	
NS1	544	F	
NS1	545	V	Contemporary Chinese CPV-associated
NS1	590	R	Less common CPV-2c variant
NS1	630	P	
VP1	131	T	VP1-specific variation
VP2	5	G	
VP2	267	Y	
VP2	324	I	
VP2	370	R	
VP2	426	E	CPV-2c determinant
VP2	447	M	Less common CPV-2c variant

Amino acids are indicated by single-letter abbreviations: V, valine; F, phenylalanine; R, arginine; P, proline; T, threonine; G, glycine; Y, tyrosine; I, isoleucine; E, glutamic acid; M, methionine. Residue numbers correspond to the amino acid position of the indicated protein.

### 3.6 *In vitro* infectivity of MDC-CPV in A72 and CRFK cells

To preliminarily evaluate the in vitroinfectivity of MDC-CPV, viral-positive samples or passaged viral supernatants were inoculated into A72 and CRFK cells. Compared with the mock-infected group, cells inoculated with MDC-CPV exhibited varying degrees of cytopathic effects, including cell rounding, detachment, and monolayer destruction. Western blot analysis revealed the expression of CPV VP2-related proteins in the infected group, whereas no corresponding signals were detected in the mock-infected group. Immunofluorescence assays further confirmed the presence of CPV antigen-positive signals within the cells of the infected group. Transmission electron microscopy revealed virus-like particles within the size range typical of parvovirus-compatible particles. These findings suggest that MDC-CPV can establish infection in A72 and CRFK cells and support the expression of viral-related proteins (Figure 5).

**Figure 5:**
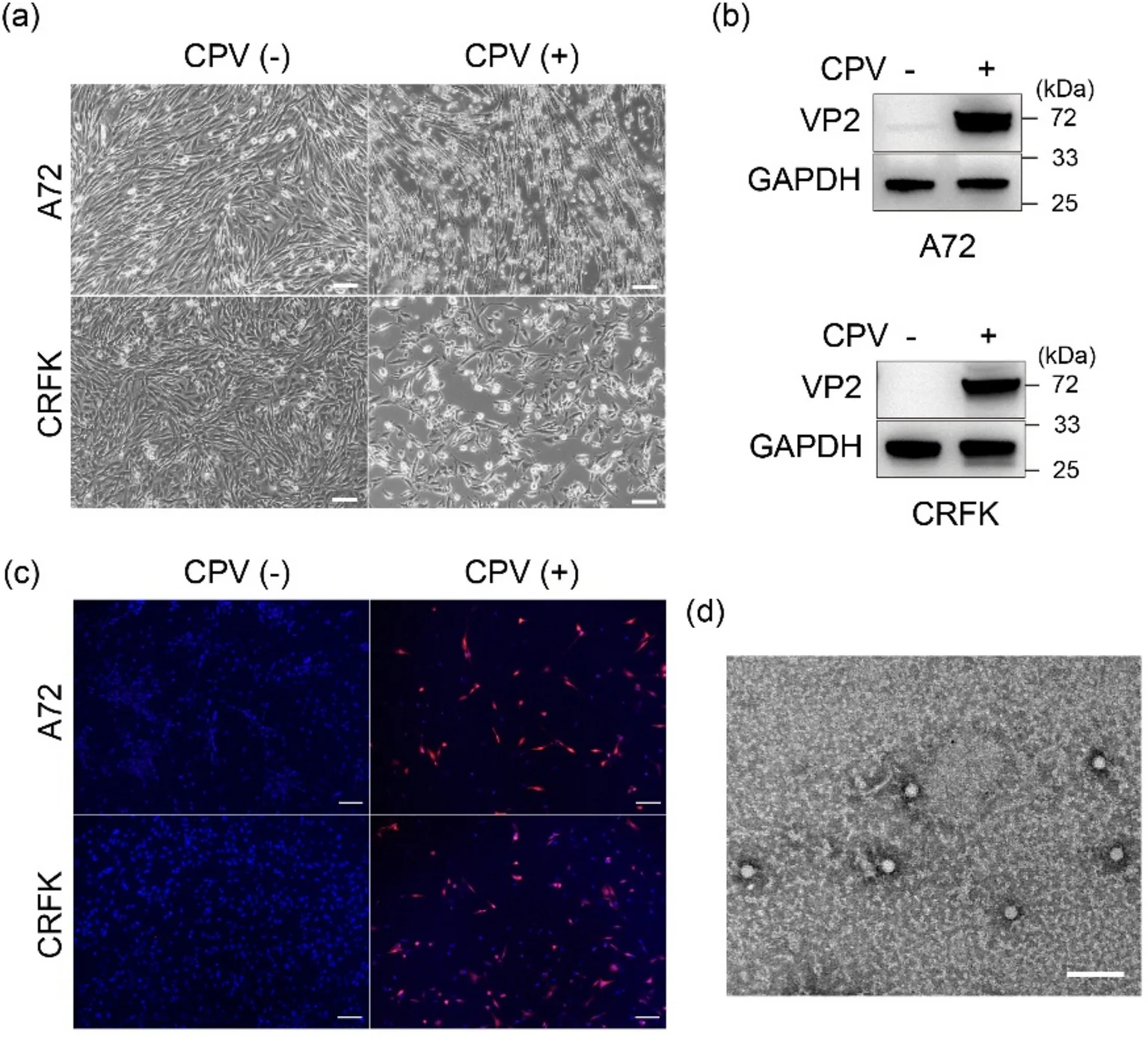
In vitro infectivity analysis of MDC-CPV. **(a)** Cytopathic effects in A72 and CRFK cells post-infection, bar = 100 μm; **(b)** detection of CPV-VP2 expression by Western blot; **(c)** immunofluorescence assay for CPV antigen detection, bar = 100 μm; **(d)** observation of virus-like particles by transmission electron microscopy, bar = 100 nm.

### 3.7 Replication kinetics of MDC-CPV in CRFK cells

To further quantitatively evaluate the replication capacity of MDC-CPV, viral DNA levels and infectious viral titers were monitored in CRFK cells over a 120-h period. Quantitative PCR analysis showed that viral DNA levels remained relatively low during the early stage of infection but increased markedly from 48 hpi onward. A progressive increase was subsequently observed at 72, 96, and 120 hpi, whereas the mock-infected controls remained at background levels throughout the observation period (Figure 6a). Consistent with the qPCR results, the infectious virus titer increased substantially during infection. The titer was low during the first 24 h, increased sharply to approximately 4.5 log_10_ TCID_50_/1 mL at 48 hpi, and subsequently reached approximately 5.3 and 5.8 log_10_ TCID_50_/1 mL at 72 and 96 hpi, respectively. The viral titer remained high at 120 hpi, with only a slight decrease compared with that at 96 hpi (Figure 6b). Taken together, the parallel increase in viral DNA levels and infectious virus titers demonstrates that MDC-CPV undergoes productive replication in CRFK cells and produces infectious progeny virus during the 120-h observation period.

**Figure 6:**
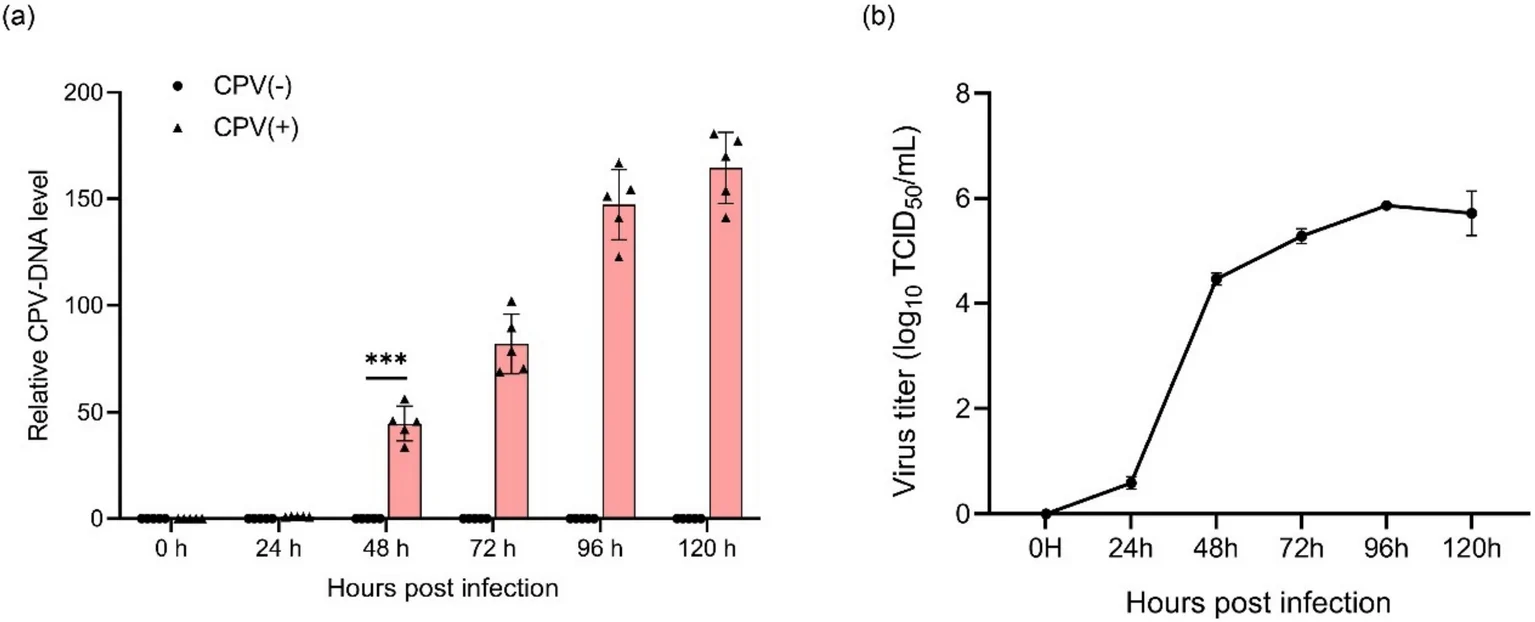
In vitro replication kinetics of MDC-CPV in CRFK cells **(a)** dynamic changes in CPV DNA levels in MDC-CPV-infected and mock-infected CRFK cells determined by quantitative PCR at 0, 24, 48, 72, 96, and 120 h post-infection **(b)** infectious viral titers at the corresponding time points determined by TCID_50_ assay and expressed as log_10_ TCID_50_/1 mL. Data are presented as mean ± SD from five independent experiments, ***p < 0.001.

## 4 Discussion

In the present study, an MDC-CPV strain derived from a clinical sample collected from a vaccinated dog in Chengdu, Sichuan Province, was molecularly characterized and shown to belong to the CPV-2c genotype. The assembled viral genome was 5,358 nt in length, and classification as CPV-2c was supported by the VP2 residue 426E together with the broader VP2 amino-acid profile and phylogenetic analysis. The genetic background of MDC-CPV is consistent with the increasing predominance of CPV-2c reported in China. A large surveillance study conducted in China during 2022–2024 identified CPV-2c as the most frequently detected genotype, accounting for 69.04% of typed strains (14). More importantly for the geographical context of the present study, a recent investigation in Sichuan Province found CPV-2c in 46 of 48 CPV-positive dogs, indicating that CPV-2c represents an important component of the currently circulating CPV population in this region (15). Nevertheless, because MDC-CPV was derived from a single clinical case, the present study cannot independently estimate the prevalence of CPV-2c in Chengdu or Sichuan.

The recombination analysis provided no robust evidence that MDC-CPV originated through recombination. Although several methods generated isolated candidate signals, no consistent breakpoint region was supported by three or more independent algorithms. Moreover, MDC-CPV remained within the CPV-2c lineage in the whole-genome, NS1, and VP2 phylogenies. The weakly supported difference in its nearest neighbor in the NS1 tree is more appropriately interpreted as insufficient phylogenetic resolution than as recombination-associated topological discordance. Therefore, recombination cannot currently be considered a demonstrated contributor to the evolutionary origin of MDC-CPV.

The VP2 residue profile of MDC-CPV, including 87 L, 101 T, 297A, 300G, 305Y, 324I, 375D, and 426E, is broadly compatible with contemporary Asian CPV-2c genetic backgrounds. Recent genomic studies in China have highlighted recurrent VP2 substitutions involving residues 87, 297, 300, 324, 375, and 426, while additional changes such as A5G, F267Y, and Q370R have been frequently reported among contemporary Asian CPV-2c lineages (14–16). These observations emphasize that CPV molecular evolution should not be interpreted solely on the basis of residue 426, as VP2 mutations can alter viral receptor affinity and antigenic characteristics (17). Furthermore, increasing evidence indicates that evolutionary changes are also present in the non-structural region. Recent Chinese CPV strains have been reported to contain recurrent NS1 substitutions, including I60V, Y544F, E545V, and L630P (14). Functional studies confirmed that these NS1 variations may modulate viral replication fitness and host-cell interaction (18). Whole-genome studies from other geographical regions have likewise identified positively selected sites in NS1, NS2, VP1, and VP2 (17). Therefore, gene-by-gene characterization of the complete coding region, rather than VP2 typing alone, provides a more informative framework for defining the evolutionary position of contemporary CPV-2c isolates.

An interesting feature of the replication curve was that viral DNA levels continued to increase toward 120 hpi, whereas the infectious titer reached its maximum at approximately 96 hpi and subsequently showed a modest decline. Although the mechanism underlying this difference cannot be determined from the current experiments, several explanations are possible. Accumulation of viral genomic DNA and noninfectious viral particles may continue after the production of infectious virions begins to plateau, while extensive cytopathic damage and depletion of viable proliferating target cells at later stages of infection may limit further production of infectious virus. Additional experiments separately quantifying intracellular and extracellular viral DNA, infectious virions, and viable cell numbers would be required to distinguish among these possibilities.

MDC-CPV was detected in a dog with a documented history of CPV vaccination. However, the detection of a field CPV-2c strain in a vaccinated animal should not be interpreted as direct evidence of vaccine failure or viral immune escape. Multiple recent case series have described CPV-2c breakthrough infections in vaccinated dogs and highlighted the multifactorial nature of protection (19). Protection against CPV can be influenced by the age at vaccination, maternal antibody interference, completion and timing of the vaccination schedule, individual immune responses, viral exposure dose, and antigenic differences between vaccine and field strains. In the present case, neutralizing antibody titers at the time of disease were unavailable and the CPV component of the administered vaccine was not sequenced. Therefore, the current data cannot establish whether antigenic differences between MDC-CPV and the vaccine strain contributed to infection. Direct comparison of MDC-CPV with the corresponding vaccine strain, together with cross-neutralization assays using vaccine-induced sera, would be necessary to address this hypothesis.

The genomic findings should also be interpreted in the context of ongoing CPV evolution. Recombination has increasingly been recognized as another potential contributor to CPV genetic diversity, and recombination events involving contemporary Chinese CPV strains have been reported (16). Consequently, comparison of whole-genome, NS1, and VP2 phylogenies together with formal recombination analysis would help determine whether the evolutionary history of MDC-CPV is concordant across the genome. Such analyses are particularly relevant when highly similar strains show discordant clustering between structural and non-structural genomic regions.

Several limitations should be acknowledged. First, this investigation was based on a single clinical case and therefore cannot provide epidemiological estimates for CPV-2c circulation in Sichuan. Second, although the assembled sequence provides extensive genomic information, the terminal regions of parvovirus genomes can be technically challenging to resolve using short-read sequencing, and independent confirmation of terminal sequences would further strengthen designation of the sequence as a complete genome. Third, the present experiments demonstrate productive infection and replication in cultured cells but do not establish pathogenicity *in vivo*. No experimental infection of dogs was performed, and the conclusions have therefore been restricted to in vitro infectivity. Fourth, the pathogen detection panel was focused on common canine pathogens did not comprehensively exclude all potential enteric co-infections. Finally, neutralizing antibody titers and the sequence of the vaccine strain administered to the affected dog were unavailable. Future studies combining broader molecular surveillance, whole-genome evolutionary analyses, vaccine-strain comparisons, neutralization experiments, and appropriately designed *in vivo* investigations will be necessary to determine the biological and epidemiological significance of contemporary CPV-2c strains (20).

## 5 Conclusion

This study reports the isolation of a canine parvovirus strain, MDC-CPV, from a clinical sample obtained from a dog with a history of vaccination. The viral genome is 5,358 nucleotides in length, with a glutamic acid (Glu/E) residue at position 426 of VP2. MDC-CPV exhibits a characteristic contemporary Asian CPV-2c amino-acid profile, including VP2 5G, 267Y, 324I, 370R and 426E. MDC-CPV is closely related to CPV-2c reference sequences from China and other East Asian regions, and demonstrates initial infectivity in A72 and CRFK cells. These findings provide a foundation for regional molecular surveillance of CPV-2c, analysis of CPV infection cases in the context of vaccine-induced immunity, and subsequent in vitrobiological studies.

## Data Availability

The complete genomic sequence of MDC-CPV has been submitted to the NCBI GenBank database under the accession number PZ493559.
